# Differential Expression Characteristics of Two Isoforms *nr5a2f* and *nr5a2m* in Gonadal Differentiation of Chinese Giant Salamanders, *Andrias davidianus*

**DOI:** 10.3390/ani15111667

**Published:** 2025-06-05

**Authors:** Dan Hu, Guanglve Li, Guohua Zou, Jiaqing Xu, Wenyin Luo, Qiaomu Hu

**Affiliations:** 1 State Key Laboratory of Mariculture Biobreeding and Sustainable Goods, Yellow Sea Fisheries Research Institute, Chinese Academy of Fishery Sciences, Qingdao 266071, China; danhu1001@163.com (D.H.); 13659644863@163.com (G.L.); x320243911@163.com (J.X.); ioop4916@163.com (W.L.); 2 Shanghai Jun Ding Fisheries Technology Co., Ltd., Shanghai 200082, China; a2406476523@163.com

**Keywords:** *Andrias davidianus*, Nr5a2, alternative splicing, differential expression

## Abstract

*Nr5a2* (nuclear receptor subfamily 5, group a, member 2) is involved in gonad development and sex hormone synthesis. In this study, the full length of *Nr5a2f* and *Nr5a2m* were obtained by *Nr5a2* variable splicing from *Andrias davidianus*, and the tissue distribution was detected. We identified *Nr5a2f* of 2455 bp and *Nr5a2m* of 2150 bp length, encoding 479 and 325 amino, respectively. We first characterized *Nr5a2f* and *Nr5a2m* gene expression in developing gonads. Results showed that *Nr5a2f* had significantly high expression in the ovary and little expression in other tissues, during the sex differentiation and sex reversal, *Nr5a2f* expression was gradually decreased in the ovary and the expression in the testis was significantly lower than in the ovary from 1 year to 6 year old. Significantly high expression was observed in the ovary and reversal ovary, while low expression was in the testis and reversal testis. While *Nr5a2m* expression exhibited the opposite profile, high expression was observed in the brain and testis. During sex differentiation and sex reversal, high expression was shown in the testis and low expression in the ovary from one year to six years old and significantly higher expression emerged in testis and reversal testis than in ovary and reversal ovary. In situ hybridization, results showed that *Nr5a2f* began to express in female undifferentiated gonads and the expression level increased from 48 dpf to 91, while *Nr5a2m* was expressed in male undifferentiated gonads. Three RNA interference sites were designed and we detected that site 293 exhibited a significant inhibitory effect in ovary cells. After *Nr5a2f* expression was inhibited by site 293, we observed that female-based gene *Nr5a2f, foxl2* and cyp19 expression were decreased, while the male-based gene *dmrt1* and *cyp17* expression was increased. These results suggested that *Nr5a2*f and *Nr5a2m* exhibited different expression patterns in the process of sex differentiation, which provided a foundation for further functional characterizations.

## Simple Summary

The aim of this research was to characterize the structure, expression and function of *Nr5a2f* and *Nr5a2m,* which was generated by alternative splicing of the *Nr5a2* gene. The expression profile suggested that *Nr5a2f* and *Nr5a2m* had different expression profiles in the gonads of females and males. *Nr5a2f* expressed significantly high in the ovary, while *Nr5a2f* in the testis. Furthermore, when *Nr5a2f* expression was inhibited, we observed that female-based gene expression was decreased, while male-based gene expression was increased.

## 1. Introduction

*Nr5a2* (nuclear receptor subfamily 5, group a, member 2, known as *LRH-1* as fetoprotein transcription factor (FTF), which belongs to the *NR5A* subfamily of nuclear receptors, is an orphan nuclear receptor and a transcriptional factor requires ligand activation*. Nr5a2* plays an important role in regulating the development, maturation and ovulation of mammals, and is a key regulator of female fertility [1,2,3]. *Nr5a2* is related to somatic cell reprogramming, embryonic development, steroid hormone production and some cancers [4]. The *Nr5a2* gene was originally identified in fruit flies. The Fushi Tarazu fragment in fruit fly was a founding member of *NR5A* [5]. Subsequently, the homologous gene has been found in mice, chickens, horses, humans and zebra fish [6]. *Nr5a2* is essential in early embryonic development, especially in maintaining the pluripotency of embryonic stem cells and endoderm formation [7]. In eutherian mammals, *Nr5a2* is mainly expressed in endoderm tissues, likely expressed in the liver, intestine and ovary [8], and high expression in the ovary [9,10]. In addition, studies found that the *Nr5a2* gene is expressed at higher levels in the ovary than in the liver or other tissues, the expression of *Nr5a2* is limited to granulosa cells and luteal cells [11]. Later studies found that *Nr5a2*^−/−^ mouse embryos died and the fertility of *Nr5a2*^+/−^ female mice decreased, which identified that *Nr5a2* plays an important regulatory role in female reproductive ability [12]. In addition, further studies have shown that *Nr5a2* up-regulates the expression of *STAR*, *Cyp11* and *Cyp19a1* genes, which promote E2 synthesis and secretion [13,14].

The Chinese giant salamander *Andrias davidianua* is the largest-tailed amphibian in the world and one of the animal resources in China [15]. However, due to its habitat destruction, overfishing and climate change, the wild resource of the giant salamander has declined dramatically and it was classified as an endangered species by the International Union for Conservation of Nature and Nature Resources in 1988 [16]. The Chinese giant salamander is believed to be a living fossil that existed before the Middle Jurassic [17]. Because the sex chromosomes of most amphibians are difficult to identify, the genes involved in the sex determination of amphibians are largely unknown. On the one hand, this is because there are multiple sex-matching types in amphibians; on the other hand, it is because the sex genes of homologous chromosomes are more likely to be converted compared to heterologous chromosomes. Amphibians have various types of genetic sex determination systems. Female (ZW) heterozygous in birds and male (XY) heterozygous in mammals can both be found in amphibians [18]. Even systems with multiple chromosomes or female OO/WO heterozygous have been reported in amphibians [19]. It can link aquatic and terrestrial organisms together and has a complex sex determination and differentiation mechanism. Therefore, it is considered to be a valuable species for studying.

Previous studies of *A. davidianus* mostly focused on the immune system [20,21,22], with little research on sex determination and sex differentiation [23,24,25]. Therefore, to study the mechanism of sex differentiation in *A. davidianus*, it is of great importance to enrich the mechanism of sex differentiation of amphibians. In this study, *Nr5a2f* and *Nr5a2m* were produced by *Nr5a2* variable splicing, which was used to produce two spliceosomes with differential expression in the gonads of males and females. This study obtained the full-length sequence of *Nr5a2f* and *Nr5a2m* from *A. davidianus*. We characterized *Nr5a2f* and *Nr5a2m* expression in different tissues, different developmental gonads and gonads from normal and sex-reversal giant salamanders by using qRT-PCR. Then, we determined their expression in undifferentiated and differentiated gonads by in situ hybridization. Furthermore, RNA interference technology was used to study the function of *Nr5a2f*. This work provides the basis for understanding the molecular mechanism of gonadal differentiation of *A. davidianus* and enriches the biological information of the *Nr5a2* gene in amphibians to further understand the mechanism of sex differentiation in amphibians.

## 2. Materials and Methods

### 2.1. Sample Collection

The *A. davidianus* were collected from Zhangjiajie Giant Salamander Breeding Base (Zhangjiajie, Hunan Province, China). This experiment followed the guidelines of the Animal Care and Use Committee of the Chinese Academy of Fishery Sciences (YSFRI-2025017). The salamanders were killed after anesthesia by MS222, samples of heart, liver, spleen, kidney, stomach, intestine, skin, muscle, lung, brain, ovary and testis tissues were collected from three species of salamanders. Gonads from male and female salamanders at the ages of 1, 2, 3, 4, 5 and 6 years old (6 years old) were collected from three individuals in each group, frozen in liquid nitrogen and then transferred to a refrigerator at −80 °C for storage until RNA extraction. The sex-reversal salamanders were prepared according to the previous reports [26]. At least three ovaries and testes from the salamander and sex-reversal salamander were collected and stored at -80 °C for RNA extraction. The developing stage gonads of 48 dpf (days post fertilization) and 98 dpf giant salamanders in the normal group were collected and then preserved in 4% paraformaldehyde (pH 7.5) to prepare tissue sections.

### 2.2. Sequence Analysis

According to the giant salamander transcriptome database from our laboratory, the sequences of *the Nr5a2* gene were obtained. By utilizing alternative promoter splicing, the giant salamander *Nr5a2* generates two splice variants, *Nr5a2f* and *Nr5a2m*. The sequence was searched in the GeneBank database using BLAST (https://blast.ncbi.nlm.nih.gov/Blast.cgi, accessed on 20 April 2025). The amino acid sequence was predicted by the software DNAman 9.0. The tertiary structure of *Nr5a2f* and *Nr5a2m* was predicted using the software SWISS-MODEL [27].

### 2.3. Quantitative Real-Time PCR

The expression of *Nr5a2f* and *Nr5a2m* genes in various tissues and developing gonads in *A. davidianus* were detected and analyzed by quantitative real-time PCR (qRT-PCR). Following the manufacturer’s instructions, total RNA was extracted from the tissue samples using TRIzol (Invitrogen, Carlsbad, CA, USA, 15596018). *EF-1α* was selected as the internal reference gene [28], and cDNA was used as a template. The primers were designed according to the gene sequences (Table 1). qRT-PCR samples from three individual tissues were used for three replicates. qRT-PCR was performed on a QuantStudio 5 real-time PCR system (APPLIED Biosystems, Foxboro, MA, USA). qRT-PCR was performed as follows: 30 s at 95 °C; forty cycles of 95 °C for 5 s, 60 °C for 30 s and 72 °C for 30 s; and then 72 °C for 5 min. Relative expression was calculated by the selected 2^−∆∆CT^ method [29]. Differences in expression were evaluated using SPSS 22.0 (IBM, New York, NY, USA). One-way ANOVA followed by Duncan’s multiple comparison tests was performed to analyze differences in Nr5a2f and *Nr5a2m*, and values of *p* < 0.05 were considered significant.

### 2.4. Fluorescence In Situ Hybridization

To detect whether *Nr5a2f* and *Nr5a2m* expression before gonadal differentiation, a fluorescence in situ hybridization probe was synthesized in the region of difference between the two spliceosome cDNA sequences of *Nr5a2*. The probe of *Nr5a2f* and *Nr5a2m* were modified with FITC and Cy3 on oligo-RNA at the 5′ end, respectively. Gonad tissues at 48 dpf and 91 dpf were collected and embedded by paraffin. The tissue paraffin blocks were treated as follows: tissue paraffin blocks were sliced by a slicer with a thickness of 4 μm, fished by a spreading machine and baked at 62 °C for 2 h. We sequentially put the slices into 2 changes of BioDewax and Clear solution, for 15 min each and then dehydrated them in 2 changes in pure ethanol for 5 min each, then put them into 85% alcohol and 75% alcohol for 5 min each and soaked them in DEPC water. Then, in situ hybridization was performed as follows: after natural cooling, the stroke circle was assembled. Protease K (20 μg/mL) was added dropwise for 40 °C digestion. The digestion time is 10 min. After washing with pure water, we performed PBS washes 3 times, for 5 min each time. We added a pre-hybridization solution and incubated it at 40 °C for 1 h. We poured out that pre-hybridization solution, added the hybridization solution containing probes and let it hybridize overnight in a constant temperature box. We washed off the hybridization solution as follows: 2 × SSC, washed at 40 °C for 10 min; 1 × SSC, washed at 40 °C for 2 × 5 min; and 0.5 × SSC was washed at room temperature for 10 min. If there are more nonspecific hybrids, formamide washing can be increased. We gently spin-dried the slice, dropped the preheated branch probe hybridization solution (60 μL) and horizontally put it in a wet box for hybridization at 40 °C for 45 min. In this process, 50 mL 2 × SSC was added to the bottom of the wet box to prevent drying. We poured off the hybridization solution and rinsed the slices with 2 × SSC, 1× SSC, 0.5× SSC and 0.1× SSC preheated at 40 °C in turn for 5 min. If there are many nonspecific hybrids, you can increase the washing time and times and adjust the concentration of formamide in the hybrid solution at this step. We added the hybridization solution containing the signal probe, and incubated it at 40 °C for 3 h at the dilution ratio of 1:400. After that, they were washed by the following SSC in turn: 2 × SSC at 40 °C for 10 min, 1 × SSC at 40 °C for 2 × 5 min and 0.5× SSC at 40 °C for 10 min. DAPI solution was dripped into the slice and incubated at room temperature for 8 min in the dark, then washed with running water and the coverslip with an anti-fade mounting medium. We used microscopy detection and collected images using Olympus-BX53 (Tokyo, Japan).

### 2.5. RNA Interference

According to the *Nr5a2f* sequence, three siRNA at different sites, nr5a2-1F-42, nr5a2-1F-139 and nr5a2-1F-293, were designed in the region of difference between *Nr5a2f* and *Nr5a2m* (Table 1). Three siRNA was modified with FAM.

According to the previous description [30], primordial ovary cells were prepared and the siRNA was transfected into the ovarian primary cell using Lipofectamine TM3000 (Thermo Fisher Scientific, Waltham, MA, USA). The cells were then incubated in opti-MEM medium (Gibco, Thermo Fisher Scientific) at 37 °C for 6 h, after which the opti-MEM medium was removed and 2 mL of DMEM including 10%PBS and P/S was added and incubated at 28 °C for 48 h. The signal of green fluorescence was detected by Olympus IX93. The RNA was extracted according to the TRIzol method for qRT-PCR to further examine sex-related gene expression.

### 2.6. Identification of Phenotypic Sex and Genetic Sex

In order to obtain the individuals with sex reversal in the previous study [26], the larvae at 55 dpf were treated with 17β-estradiol or exposed to temperatures of 28 °C until eight months after fertilization. The genetic sex of the giant salamander used was identified using the female-specific marker *adf431* developed in our laboratory, and tissue sections and microscopes were used to identify the biological sex of the giant salamander samples. The reaction mixture consists of 100 ng genomic DNA, 12.5 μL 2× Master Mix (Tsingke, Nanjing, China), 0.5 ‌μL of each primer (10 μm) and double-distilled water, with a final volume of 25 ‌μL. The reaction conditions were 95 °C for 5 min, 33 cycles of 15 s at 94 °C, 15 s at 60 °C, 72 °C for 30 s and 5 min at 72 °C. If the phenotypic sex and genetic sex are consistent, there will be no sex reversal. If the phenotypic sex and genetic sex are different, the genetic sex is male and the phenotypic sex is female, then this individual is regarded as a sex-reversed male (RM) individual. If the sex is female and the genotype is male, then this individual is regarded as a sex-reversed female (RF) individual.

## 3. Results

### 3.1. Sequence Analysis of Nr5a2f and Nr5a2m

**Figure 1:**
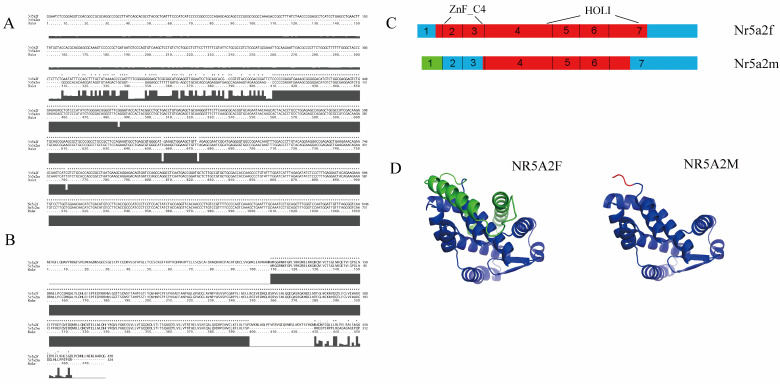
***Nr5a2* spliceosome sequence and structure model diagram.** (**A**) Nucleotide sequence analysis; (**B**) amino sequence analysis of *Nr5a2f* and *Nr5a2m*; (**C**) *Nr5a2f* and *Nr5a2m* structure. Blue color indicates untranslated region in cDNA. Red color indicates exon. Blue color indicates non-translated region. The green color indicates 5′ end untranslated region in Nr5a2m, which was different from Nr5a2f; (**D**) tertiary structure diagrams of *Nr5a2f* and *Nr5a2m* proteins. * indicated same amino acid.

By alternative splicing, the giant salamander *Nr5a2* generates two splice variants, *Nr5a2f* and *Nr5a2m*. The full length of *Nr5a2f* was 2455 bp, and the full length of *Nr5a2m* was 2150 bp (Figure 1A). *Nr5a2f* encodes 479 amino acids, and *Nr5a2m* encodes 325 amino acids (Figure 1B). *Nr5a2f* encodes the full-length transcript, whereas *Nr5a2m* loses the first exon due to alternative promoter usage and initiates transcription at the first intron (Figure 1C). The online prediction software predicted the tertiary structure of proteins encoded by *Nr5a2f* and *Nr5a2m*, as shown in the figure (Figure 1D).

### 3.2. Expression of Nr5a2f and Nr5a2m in Different Developing Gonads and Sex Reversal

**Figure 2:**
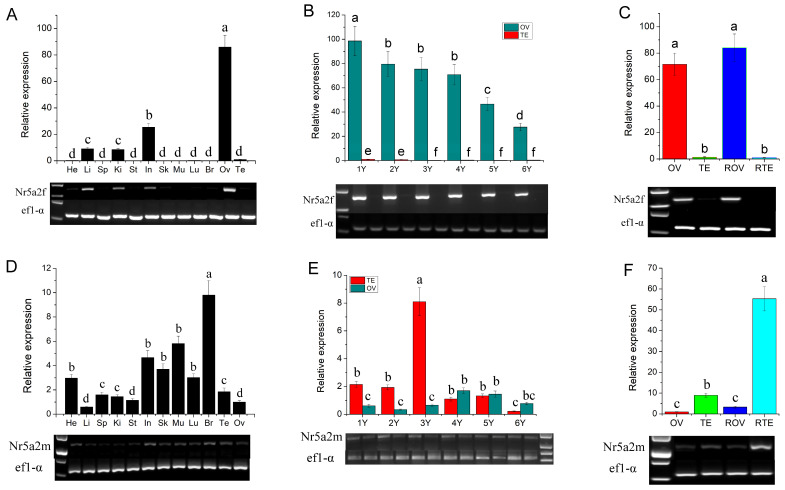
Expression of *Nr5a2f* and *Nr5a2m* in different stages of developmental and sex reversal gonads. (**A**) Expression analysis of *Nr5a2f* in various tissues; (**B**) expression analysis of *Nr5a2f* in developing gonads; (**C**) expression analysis of *Nr5a2f* in sex reversal gonads of *A. davidianus*; (**D**) expression analysis of *Nr5a2m* in various tissues; (**E**) expression analysis of *Nr5a2m* in developing gonads; (**F**) expression analysis of *Nr5a2m* in sex reversal gonads of *A. davidianus.* ANOVA followed by Duncan’s multiple comparison tests. Different letters differed with statistical significance (*p* < 0.05). He, heart; Li, liver; Sp, spleen; Ki, kidney; St, stomach; In, intestine; Sk, skin; Mu, muscle; Lu, lung; Br, brain; Ov, ovary; Te, testis; ROV, sex reversal ovary; RTE, ex reversal testis.

Expression levels of *Nr5a2f* and *Nr5a2m* in different tissues, developing gonads and sex-reversal gonads, were detected using qRT-PCR and RT-PCR. Results of qRT-PCR and RT-PCR have high consistency. *Nr5a2f* was expressed high in the ovary, low in the intestine, liver and kidney, and little expression in other tissues (Figure 2A). During gonadal development, *Nr5a2f* expression was significantly higher in the ovary than in the testis, with expression levels gradually decreasing from 1 to 6 years (Figure 2B). The expression level of *Nr5a2f* in the sex-reversal ovary is significantly higher than in the testis and similar to that in the normal ovary (*p* < 0.05; Figure 2C). Expression of *Nr5a2m* was observed in various tissues and significantly higher expression levels were detected in the testis than that in the ovary (*p* < 0.05; Figure 2D). Expression of *Nr5a2m* in the testis was significantly higher than in the ovary from one year to three years, while, from four years to six years, no significant difference was observed between the ovary and testis (Figure 2E). The expression of *Nr5a2m* in sex-reversal testis was significantly higher than that in normal testis and ovary (*p* < 0.05; Figure 2F).

### 3.3. Expression of Nr5a2f and Nr5a2m Identified by In Situ Hybridization

**Figure 3:**
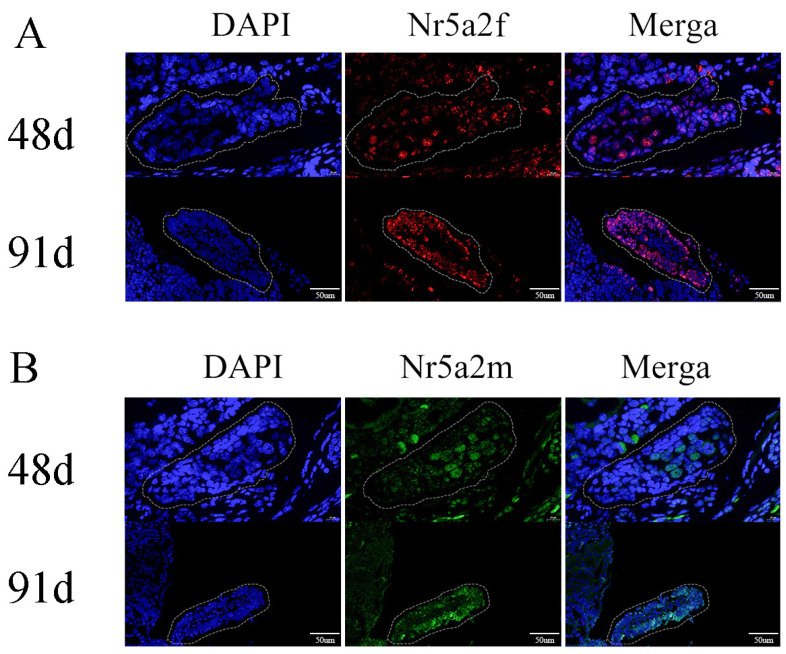
Expression of *Nr5a2* (**A**) and *Nr5a2m* (**B**) in gonads detected by in situ hybridization. (**A**) Expression of *Nr5a2f* in undifferentiated gonads on 48 d and 91 d; (**B**) expression of *Nr5a2m* in undifferentiated gonads on 48 d and 91 d. The dashed box shows the early gonads.

To detect whether *Nr5a2f* or *Nr5a2m* expressed before gonad differentiation, in situ hybridization was conducted to identify the expression of *Nr5a2f* and *Nr5a2m*. In a previous study, we determined that the gonad of *A. davidianus* began to differentiate about 98 days post fertilization, and the female-specific marker was explored to determine the genetic sex of *A. davidianus.* In situ hybridization results showed that *Nr5a2f* began to express before gonadal differentiation in female *A. davidianus*, with its signal strength increased from 48 dpf to 91 dpf (Figure 3A). In contrast, *Nr5a2m* starts to express before gonadal differentiation in male *A. davidianus* (Figure 3B).

### 3.4. RNA Interference

**Figure 4:**
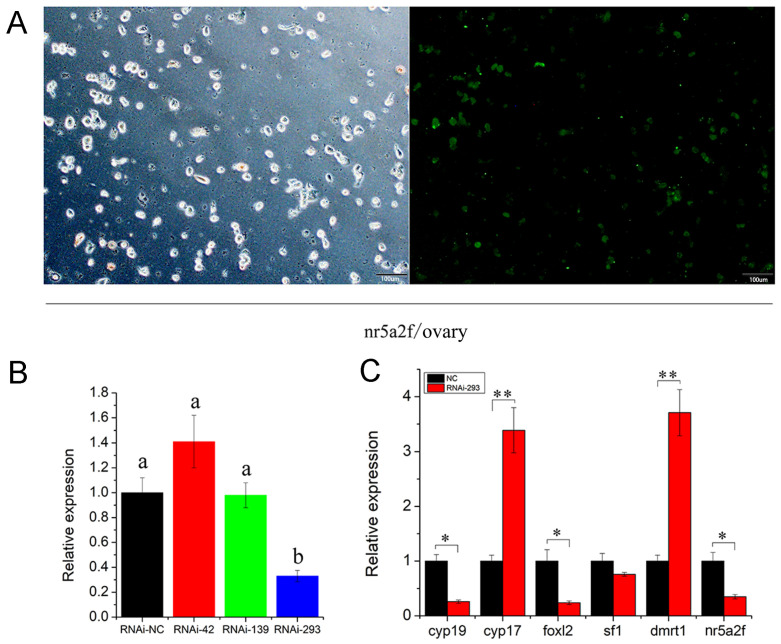
Inhibited expression of *Nr5a2f* affected the transcription of sex-related genes in *A. davidianus* gonad. (**A**) RNAi of *Nr5a2f* transfected into ovary primary cells of *A. davidianus*; (**B**) expression profile of *Nr5a2f* after Nr5a2f/siRNA treated at different interference sites; (**C**) expression profile of sex-related gene after treated with RNAi-293 sites. Data are the mean ± sd. * above columns indicate significant differences among groups. (*p* < 0.05), ** indicate extremely significant difference (*p* < 0.01). ANOVA followed by Duncan’s multiple comparison tests. Different letters differed with statistical significance (*p* < 0.05).

To examine the function of *the Nr5a2f* gene in the gonad, three RNA interference (RNAi) sites were designed based on the differential regions of *Nr5a2f* and *Nr5a2m*. Liposome 3000 was used to transfect RNAi to primary ovarian cells from one-year-old giant salamanders. The transfection efficiency was exhibited at 50% after 48 h (Figure 4A). The RNAi effects were evaluated using qPCR, which revealed that the RNAi-293 site had the best inhibition effect (Figure 4B). The expression profile of sex differentiation-related genes was detected after *Nr5a2f* gene expression interfered. The results showed that the expression of *Nr5a2f*, *Foxl2* and *Cyp19* genes were significantly down-regulated (Figure 4C; *p* < 0.05), while the expression of *Dmrt1* and *Cyp17* genes were remarkly up-regulated (*p* > 0.05). No significant difference was observed in the expression of *sf1* gene (*p* > 0.05). The results indicate that the *Nr5a2f* gene regulated the expression of sex differentiation-related genes and participated in the regulation of the sex differentiation process of giant salamanders.

## 4. Discussion

Among different *A. davidianus* tissues, the highest expression level of *Nr5a2f* was observed in the ovary, followed by the intestine, and a low level was found in other tissues. Mendelson et al. [31] showed that *Nr5a2* was widely expressed in mammalian tissues, with high expression observed in granulosa cells from primordial follicles to preovalatory follicles and in luteal cells. The relative expression of *Nr5a2m* was evaluated by qRT-PCR. The results showed that the relative expression of *Nr5a2m* in *A. davidianus* was the highest in the brain, and the expression level in the testis was significantly higher than that in the ovaries. As gonadal development progressed, *Nr5a2m* gene expression had no significant difference in male and female gonads from four years to six years. The *Nr5a2* gene is an important member of the nuclear receptor superfamily and plays an important role in growth development and cell differentiation. In this study, a higher expression of *Nr5a2f* was detected in the ovary than in the testis, which indicates that *Nr5a2f* played an important role in ovarian development. Additionally, we found that the expression of *Nr5a2f* increased during the process of male-to-female sex reversal, with significantly higher expression levels in the ovary and sex-reversed ovary compared to the testis and sex-reversed testis. The above results indicate that the high expression of *Nr5a2f* in the ovary confirms the crucial role in ovarian differentiation and development. Similar expression patterns have been observed in other species [32,33,34]. For instance, Boerbom found that the *Nr5a2* gene in horses is highly expressed in gonadal tissues, indicating ovary-specific expression [6]. Granulosa cell proliferation in *Nr5a2-*specific knockout mice was impaired, disrupting the normal processes of E2 synthesis and follicle development, resulting in the mice being unable to ovulate normally [35,36]. Further studies showed that the expression of *Nr5a2* is restricted to granulosa cells at various stages of ovarian follicle development, and its downstream target genes were almost all involved in follicular development processes, including genes regulating granulosa cell proliferation, E2 synthesis and ovulation [35,37]. In situ hybridization results determined that *Nr5a2f* began to express before gonadal differentiation in female *A. davidianus*, with its expression level increased from 48 dpf to 91 dpf. In contrast, *Nr5a2m* starts to express before differentiation in male *A. davidianus*, which indicates that the *Nr5a2f* gene plays a vital role in the ovary of *A. davidianus*, but the role in the testis is not obvious.

Sex differentiation in amphibians is complex, probably due to their typical two-stage life cycle. Sex differentiation is one of the hot topics in aquatic animal research, *Cyp19* and *foxl2* are key genes in estrogen synthesis, which play a significant role in the sex differentiation and development of animals, particularly in the differentiation and functional maintenance of ovaries [38]. In the process of sex determination and gonad differentiation, *cyp19a*, *Dmrt1*, *Foxl2* and *Nr5a2* genes interact with each other through a complex regulatory network to control gonad development direction. The genetic studies showed that three developmental genes, *Foxl2*, *cyp19a* and *Dmrt1,* play an indispensable role in the gonad development of the Chinese giant salamander [39,40,41,42]. *Dmrt1* plays a key role in male development in mammals, birds, amphibians and bony fish [43,44,45]. *Foxl2* is considered to be a highly conserved gene in vertebrates and is involved in almost all stages of ovarian development [46,47,48,49,50,51,52]. *Cyp19a* plays an important role in vertebrate sex determination and differentiation and is mainly designed to participate in the regulation of estrogen in ovarian differentiation [53,54,55,56]. In this study, when nr5a2f expression was inhibited, female-based gene expression was decreased, while male-based gene expression was increased. The results suggested that nr5a2f regulated the female base genes foxl2 and cyp19a to affect sex differentiation. Above all, we cloned the full-length sequence of *Nr5a2f* and *Nr5a2m*, which were produced through alternative splicing. *Nr5a2f* and *Nr5a2m* were expressed in various tissues, and the expression of *Nr5a2f* in the ovary was significantly higher than that in the testis, whereas the expression of *Nr5a2m* in the testis was higher than that in the ovary. *Nr5a2f* began to express before the gonadal differentiation in female *A. davidianus*, and *Nr5a2m* started expressing before the differentiation in males. When RNAi-293 was applied, the expression of *Nr5a2f*, *Foxl2* and *Cyp19* genes were significantly down-regulated, and the expression of *Dmrt1* and *Cyp17* genes were significantly up-regulated. The results indicated that the *Nr5a2f* gene regulates the expression of genes related to sex differentiation and was involved in the sex differentiation process of *A. davidianus*. Further study is needed to determine whether it is essential for ovarian development.

## 5. Conclusions

In this study, we characterized *Nr5a2f* and *Nr5a2m* gene expression in various developing gonads and in sex reversal. In situ hybridization was used to detect the expression of *Nr5a2f* and *Nr5a2m* in undifferentiated gonads. Differential expression of *Nr5a2f* and *Nr5a2m* genes were studied and revealed an important role of *Nr5a2f* in gonad differentiation. Furthermore, when we inhibited the expression of *Nr5a2f*, we found that the sex-related gene expression significantly changed. Further study in the future should be carried out to unveil the function of Nr5a2 and the mechanism of alternative splicing. A thorough understanding of the sex differentiation mechanism of *A. davidianus* can not only enhance the understanding of amphibians, but also improve the Chinese giant salamander farming industry.

## Figures and Tables

**Table 1:** Sequence of the primers used in this study.

Name	Sequence	Utilizations
Sf1-S	CAGTGGATTATGACAGAAGTCCGTA	qRT-PCR
Sf1-A	GGTCTCGGGAATGTCAGGGTA	qRT-PCR
foxl2-S	GGTAGCCGTAGCTGTCGCTG	qRT-PCR
foxl2-A	AGAACAGCATCCGCCACAAC	qRT-PCR
cyp19a-S	CCTTCATACGGACGGCTTGT	qRT-PCR
cyp19a-A	CAGATTAGAAGCAGGACACCCATA	qRT-PCR
dmrt1-A	GCCATTGGTTGCCTGATTG	qRT-PCR
dmrt1-S	ACCAGGTGGCAGTGGCTTC	qRT-PCR
cyp17-S	GCAGCGTCTCCTTGATGGTC	qRT-PCR
cyp17-A	ACAGACGGAGGTGAGGACGAC	qRT-PCR
nr5a2mS:	GAGCCTGCACAGCGAGAGG	qRT-PCR
nr5a2mA:	CCCACGCTCAGGCACTTCT	qRT-PCR
nr5a2fS:	CGGCATGGAGGTTGGAATC	qRT-PCR
nr5a2fA:	CAGGCAGGTGTAGTGCTTGTTATT	qRT-PCR
ef1-α-F	GGACAGACCCGTGAACATGC	Internal control
ef1-α-R	CTTCCTTAGTGATCTCCTCGTAGC	Internal control
adf431a	TCCAGAATGAAGTCCTGGCCT	Sex identification
adf431s	CGAGCCTCCATTGTGCCTT	Sex identification
nr5a2-1F-42S	AGCACGCCUACGCUGAUUUTT	RNAi/5-end, modify with FAM
nr5a2-1F-42A	AAAUCAGCGUAGGCGUGCUTT	RNAi
nr5a2-1F139S	GAGCCUGAACUUUAUGGUATT	RNAi/5-end, modify with FAM
nr5a2-1F139A	UACCAUAAAGUUCAGGCUCTT	RNAi
nr5a2-1F293S	GGCUACCCCUCUUCUGAAUTT	RNAi/5-end, modify with FAM
nr5a2-1F293A	AUUCAGAAGAGGGGUAGCCTT	RNAi
NC-S	UUCUCCGAACGUGUCACGUTT	Control/5-end, modify with FAM
NC-A	ACGUGACACGUUCGGAGAATT	Control
NR5A2F	CCAGACGGCGCAGAATACGAAAAAGGAAGAGGCCAGAGACAGGAGCTTGA	FISH, modify with FITC
NR5A2M	CCGCTTTCATCTCGGGGGCTTCCTTCTACTTTCTGGCTCATCCTCTCGCT	FISH, modify with Cy3

## Data Availability

The data presented in this study are available in the article.
